# The Study of an Ultraviolet Radiation Technique for Removal of the Indoor Air Volatile Organic Compounds and Bioaerosol

**DOI:** 10.3390/ijerph16142557

**Published:** 2019-07-17

**Authors:** Chao-Yun Liu, Chao-Heng Tseng, Huang-Chin Wang, Chuan-Fa Dai, Yi-Hsuan Shih

**Affiliations:** 1 Institute of Environmental Engineering and Management, National Taipei University of Technology, Taipei 10608, Taiwan; 2 Union Professional Group of Architecture, Taipei 10608, Taiwan

**Keywords:** ultraviolet radiation, bioaerosol, formaldehyde, total volatile organic compounds, indoor air quality

## Abstract

This study examined the use of high dosages of ultraviolet germicidal irradiation (UVGI) (253.7 nm) to deal with various concentrations of air pollutants, such as formaldehyde (HCHO), total volatile organic compounds (TVOC), under various conditions of humidity. A number of irradiation methods were applied for various durations in field studies to examine the efficiency of removing HCHO, TVOC, bacteria, and fungi. The removal efficiency of air pollutants (HCHO and bacteria) through long-term exposure to UVGI appears to increase with time. The effects on TVOC and fungi concentration were insignificant in the first week; however, improvements were observed in the second week. No differences were observed regarding the removal of HCHO and TVOC among the various irradiation methods in this study; however significant differences were observed in the removal of bacteria and fungi.

## 1. Introduction

Airborne microorganisms, such as bacteria, fungi, fungal toxin viruses, and actinobacteria can cause allergies, irritation, and contagious diseases [1]. Ultraviolet (UV) light can be used for the removal of these pollutants, with the sterilization efficiency determined by the wavelength of the light applied. The World Health Organization defined four ranges of ultraviolet light as follows: VUV (100–200 nm), UV-C (200–280 nm), UV-B (280–320 nm), and UV-A (320–400 nm). UV-A and UV-B share a similar sterilization mechanism, which involves breaking single strands of DNA and destroying the cell membrane in microorganisms [2,3]. However, many organisms possess a repair mechanism capable of combatting these effects. The sterilization mechanism of UV-C comprises both physical and biochemical processes. After absorbing the UV light, DNA thymine can cause two neighboring thymines to cross link and may form pyrimidine dimers and thymine dimers, which will be able to interfere with the subject’s DNA and eventually lead to cell death. [4,5,6,7].

Ultraviolet germicidal irradiation (UVGI) is generated using low pressure mercury vapor. Within the UV-C band of the electromagnetic spectrum (100–290 nm), more than 90% of the output irradiation is at a wavelength of 253.7 nm [8,9]. UVGI has been applied in disinfection and sterilization for treating water, disinfecting surfaces, and preventing the spread of disease through the air [3,10]. However, most previous studies have focused on the mechanisms involved in the disinfection of microorganisms and appropriate dosages. Few researchers have addressed the issues of removal efficiency, irradiation duration, or the methods of irradiation used for particular air pollutants.

UVGI is generally applicable in three areas, as follows: Inside the ducts used for mechanical ventilation, return air units, and any indoor area [4]. The DNA of contagious airborne pathogens is damaged by the energy of UVGI light, which interferes with its duplication, rendering the organisms noncontagious. However, the likelihood that this damage will lead to cell death varies according to the type of organism and its exposure to UVGI [11]. Unfortunately, UVGI can cause erythema, photokeratitis, and conjunctivitis. The American Conference of Governmental Industrial Hygienists (ACGIH) established a threshold limit value (TLV), the dose to which a worker can be exposed for eight hours a day, of 40 h per week for a working lifetime without adverse health effects, as a guideline for avoiding skin and eye injuries. The TLV is expressed as a dose (J/cm^2^), the product of irradiance (in W/cm^2^) and time (in seconds). For 254 nm, the dose limit is 6.0 mJ/cm^2^. Thus, a worker can be exposed to an irradiance of 60 mW/cm^2^ for 1 s or to 0.21 μW/cm^2^ for eight hour [12,13]. Upward irradiation is commonly used to prevent or minimize exposure [14,15]. In such cases, organisms must be directed into upper areas by forced air (mechanical) or natural ventilation (buoyancy-driven) to facilitate disinfection by UVGI [11].

The mechanism by which UV light removes air pollutants is photochemical dissociation, which occurs at wavelengths ranging between 100 and 1000 nm. This process involves the absorption of photons by molecules, resulting in the excitation of their electrons enabling them to jump from low- to high-energy states. Moving from the ground state to an excited state destabilizes the photons, resulting in the release of light or heat or a reaction with other molecules when the molecules return to their original ground state. Excited electrons can break the chemical bonds, thereby altering the physical and chemical properties of the molecule [16,17].

The photons associated with UV light of shorter wavelengths are more energetic, making them better able to remove air pollutants. In the direct photolysis of organic pollutants, Burrows et al. [18], Lin [19], and Ao et al. [20], demonstrated the ability of UV-C in breaking down formaldehyde and toluene molecules. However, the photolysis of 4-nitrophenol could not be achieved using UV light at a wavelength of 365 nm due to the **C-N, C-C, C=C, C-H, and C-O** bonds within the structure. The **C-N** bond (maximum wavelength of 392.7 nm) can be broken by UV light at a wavelength of 365 nm; however, the maximum wavelength of the other bonds range from 196.1 nm to 346.1 nm, rendering the UV light too weak for the direct photolysis of 4-nitrophenol [21].

The elimination of air pollutants by UVGI at wavelengths less than 290 nm involves direct photolysis, in which molecules that absorb light energy enter a chemically active state that breaks their chemical bonds, resulting in further dissociation reactions or the promotion of reactions with other substances [20]. In Shie et al. [22], it was indicated that UV light of shorter wavelengths is more efficient for the removal of formaldehyde (HCHO). The means by which photolysis occurs is determined by the chemical bonds in the molecules as well as the energy provided by UVGI. The efficiency of UVGI for the removal of pollutants is also determined by the dosage of UV light, the number of UV light lamps in a given area, and the method of irradiation, as well as the relative humidity (RH), temperature, air flow, and mixing of air in the environment [23,24].

A review of relevant literature revealed that most research on UV lighting techniques has focused on sterilization mechanisms and the quantity of disinfectants on the surfaces of microorganisms, rather than on the efficiency with which air pollutants are removed. This study examined the efficiency of using high dosages of UVGI (253.7 nm) for the removal the air pollutants (HCHO and TVOC (Total Volatile Organic Compounds)) of various concentrations from environmental chambers under various levels of relative humidity. We also conducted field tests to investigate the efficiency of various UVGI irradiation methods (direct irradiation overnight, upward irradiation, and upper space irradiation) in the removal of indoor air pollutants over various periods of time. In addition, the Taiwan Environmental Protection Administration (Taiwan EPA) issued the Indoor Air Quality Standard according to the Indoor Air Quality Management Act in 2012. This standard contains nine indoor air pollutants, CO_2_, CO, HCHO, TVOC, PM_2.5_, PM_10_, O_3_, total fungi, and total bacteria. The standard values for HCHO, TVOC, fungi, and bacteria are 0.08 ppm (one-hour average), 0.56 ppm (one-hour average), 1000 CFU m^−3^, and 1500 CFU m^−3^. UVGI are tested for controlling indoor air pollutant concentrations to meet Taiwan IAQ standards.

## 2. Materials and Methods

### 2.1. UVGI Experiments in Environment Chambers

**Figure 1:**
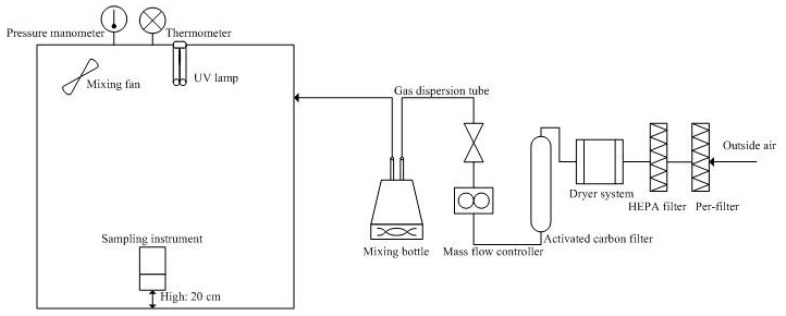
Schematic diagram of the UVGI experiment system.

This study employed UVGI lamps (XH-20, 20W-UVC) containing low-pressure mercury-vapor for the emission of short-wave UV radiation (253.7 nm). The lamps are 13 cm long with a radius of 1.9 cm. The UVGI lamp has been treated to block wavelength at 180 nm. The batch experiment was designed to explore the effects of initial concentration and relative humidity on the removal efficiency of UVGI at zero air exchange rate. The size of the stainless steel and glass chamber was L1.0 m × W1.0 m × H1.5 m (1.5 m^3^). Prior to the experiments, 75% ethanol was sprayed on the inner walls of the chamber, which were then wiped with water (>60 °C) to promote vaporization. We tested the chamber for leakage and ensured that background concentrations of HCHO and TVOC were lower than 0.05 ppm by setting instruments for monitoring air quality at 20 cm above ground level. The UVGI luminaire was installed 1 m above the air quality monitor instruments. Initially, the chamber was sampled over a 12 h period to evaluate air quality without air exchange (ACH = 0), as shown in Figure 1.

HCHO was produced by dissolving 4 g of paraformaldehyde in 200 g of the distilled water. The paraformaldehyde solution bottle was connected with zero gas to produce formaldehyde gas by aeration (10 L/min). Formaldehyde was injected into the chamber through a pipe until the concentration reached the level required for each experiment. TVOC was produced by spraying volatile organic compounds (solvent-based paint) onto a 15 cm × 15 cm stainless steel plate and then placed in an acrylic container. The container was purged with zero gas to create diluted VOC in the chamber. By injecting various ratios of zero gas, the required concentration of TVOC was obtained. The relative humidity of the chamber was controlled by injecting various ratios of dry zero gas. The conditions used in these experiments were as follows: High (1.0 ± 0.1 ppm) and low (0.5 ± 0.1 ppm) initial concentrations of HCHO, high (3.0 ± 0.1 ppm) and low (1.4 ± 0.1 ppm) initial concentrations of TVOC, and high (70 ± 2%) and low (40 ± 2%) relative humidity (RH) of both HCHO and TVOC. The experiments were repeated twice for each test condition to confirm the reproducibility of the results. The replicate tests of HCHO and TVOC show that all errors were less than ± 5%. For bacteria and fungi measurements in field tests, duplicate samples were taken for each location. Either 2 or 4 different locations in each site were tested (depending on the size of the site). Reported results of bacteria and fungi measurements were the average results of all duplicate samples in all locations for each site. The replicate tests of bacteria and fungi show that all the errors were less than ± 10%.

### 2.2. UVGI Experimental in Field Studies

**Figure 2:**
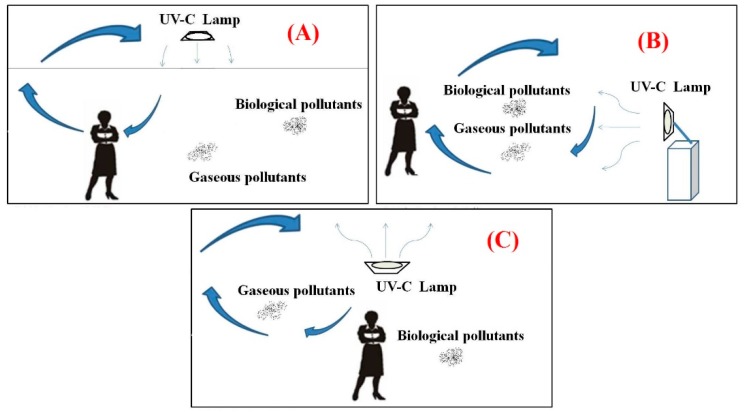
The configuration of three types of UVGI luminaire in field tests: (**A**) Upper space irradiation, (**B**) direct irradiation overnight, and (**C**) upward irradiation.

Four sites were selected for the field tests in this study, as follows: An underground parking lot, a kitchen waste area, an integrated *traditional Chinese* and *western medicine* clinic (Clinic A), and a *medical cosmetics clinic* (Clinic B). Samples (two each morning and two each afternoon) were obtained from each site over the period from 15 November 2009 to 20 April 2010 to determine the removal efficiency of UVGI. Test subjects included TVOC, HCHO, bacteria, and fungi. Background concentrations were measured prior to the initiation of UVGI irradiation. Since it is more difficult to control the environmental conditions of field test, this study omits the natural decline of HCHO and TVOC. The UVGI irradiation plans implemented in the test sites are presented in Table 1. The direction of UVGI irradiation is shown in Figure 2.

A Formaldemeter htv-m instrument (PPM Technology, Caernarfon, UK) was used to measure HCHO, which had been placed in a calibration tube in the test environment for at least one hour to balance its temperature with that of the environment location, with references to the accompanying temperature and formaldehyde concentration table to calibrate the instrument. A ppbRAE-3000 VOC monitor (**RAE Systems Inc.**, **Sunnyvale, CA**, USA) was used to measure TVOC. To ensure the accuracy of experiment data, zero-point calibration was performed using an activated carbon tube and span calibration using 10 ppm of isobutylene (C_4_H_8_) gas, with a calibration error less than 10%. Table 2 lists the specifications of the instruments used for measuring air pollutants.

Bioaerosol sampling was performed using an Andersen one-stage sampler (Thermo electron corporation, Waltham, MA, USA) with 200 holes and air throughput of 28.3 L min^−1^. Sampling methods for bacteria and fungi were based on standards E301.12 C and E401.12 C, as set out by the Environmental Protection Administration (EPA) of Taiwan [25,26]. Collected fungi were placed on malt extract agar (MEA) medium and incubated at 25 °C for 3 days. Collected bacteria were placed on tryptic soy agar (TSA) medium and incubated at 30 °C for 1 day. Two duplicate samples of bacteria and fungi were also measured. The difference in flow rate (28.3 L/min), as measured using a hot wire anemometer before and after sampling, was maintained at <10% (±2 L/min). The number of colony-forming units per cubic meter of air (CFU m^−3^) was calculated using Equation (1), as follows:

(1) $$Bioaerosol\;conc.\;\left(CFU\;m^{-3}\right)\;=\;\frac{\left[\left(Avg\;count\;for\;stage\;0\right)+\left(Avg\;count\;for\;stage\;1\right)\right]}{flow\;rate\;\left(28.3\;l/min\right)\times Sampling\;time\;\left(L\right)}\times 1000\frac{L}{m^{3}}.$$

### 2.3. UVGI Removal Efficiency of Air Pollutants

Removal efficiency was calculated using Equation (2). Equation (2) is used to calculate the pollution removal efficiency in the chamber; however, the deposition and adsorption of air pollutants proved difficult to estimate in practical applications. Thus, the calculation of UVGI removal efficiency did not include the natural rate of decline in the percentage of air pollutants that can be expected to occur in the field, as shown in Equation (3):

(2) $${UVGI\;removaleff}_{test\;chamber}\left(\%\right)=\frac{\left[\left(C_{{UVGI}_{before}}-C_{{UVGI}_{after}}\right)/C_{{UVGI}_{before}}\right]-\left[\left(C_{O}-C_{i}\right)/C_{O}\right]}{1-\left[\left(C_{O}-C_{i}\right)/C_{O}\right]}\times 100\%$$

(3) $${\mathrm{UVGI}\;\mathrm{removaleff}}_{\mathrm{field}}\left(\%\right)=\frac{\left(C_{{UVGI}_{before}}-C_{{UVGI}_{after}}\right)}{C_{{UVGI}_{before}}}\times 100\%$$

where *C_UVGI before_* is the concentration of air pollutants before prior to the application of UVGI and *C_UVGI after_* is the concentration of air pollutants after using UVGI. The test time for the natural decline experiment in the chamber lasts for 12 hours. The value *C_O_* is the initial concentration of air pollutants by the natural decline test and *C_i_* is final concentration of air pollutants by natural decline.

### 2.4. Measurement of UV Irradiance and Calculation of Dosages

**Figure 3:**
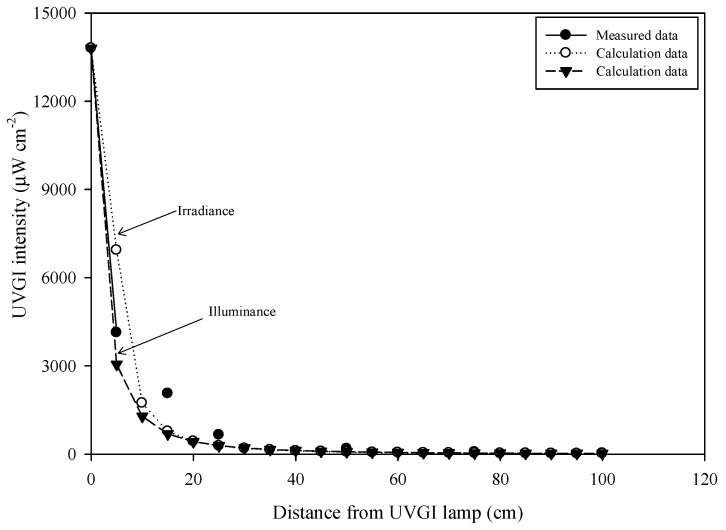
Calculated data and measured data of UVGI intensity at various distances from the UVGI lamp.

We calculated the radiation view factor to estimate UVGI intensity [27], as shown in Equation (4). The intensity of UVGI was measured using an UV-C light meter (Lutron electronic enterprise CO., LTD.; model: UVC-254) with a 254 nm sensor in order to quantify the difference between the calculated and measured data associated with UVGI intensity at various distances from the lamp, as shown in Figure 3. The measured data is the illuminance of the surface on the lamp by UV-C light meter, which is 13,800 μW/cm^2^ at 0 cm. To calculate the electron volts at different distances from the lamp source, the illuminance value (μW/cm^2^) of different distances must be measured first, then the illumination value is exchanged to electron volts and multiplied by molecular surface area. The result showsed how much energy is needed to destroy the molecular bond.

(4) $$F=\frac{l/r}{\pi (x/r)}\left[\begin{array}{l} \frac{1}{l/r}\left(\tan^{-1}\frac{l/r}{{\left(x/r\right)}^{2}-1}\right)-\tan^{-1}\left(\sqrt{\frac{(x/r)-1}{(x/r)+1}}\right)+\frac{\left(1+{\left(x/r\right)}^{2})+{\left(l/r\right)}^{2}-2(x/r\right)}{\sqrt{\left((1+{\left(x/r\right)}^{2})+{\left(l/r\right)}^{2})((1-{\left(x/r\right)}^{2})+{\left(l/r\right)}^{2}\right)}}\\ \tan^{-1}\left(\sqrt{\frac{\left((1+{\left(x/r\right)}^{2})+{\left(l/r\right)}^{2})((x/r)-1\right)}{\left((1-{\left(x/r\right)}^{2})+{\left(l/r\right)}^{2})((x/r)+1\right)}}\right) \end{array}\right]$$

where *x* is distance from the lamp (cm), *l* is length of the lamp segment (cm), and *r* is the radius of the lamp (cm).

Irradiation intensity at any given point is determined according to surface irradiation intensity (I_sur_), as shown in Equation (5):

(5) $$I_{sur}\;(\mu Wcm^{-2})=\frac{E_{UV}F}{2\pi rl}$$

where *I_sur_* is the UV intensity at *x,y,z* point, *E*_UV_ is the power output of the lamp (W cm^−2^), *F* is the radiation view factor, *r* is the radius of the lamp, and *l* is the length of the lamp segment (cm).

The efficiency of UVGI in removing air pollutants depends heavily on whether the energy it generates is sufficient to break the chemical bonds in question. We therefore used the Planck equation (Equation (6)) to derive the photon energy of UVGI at various wavelengths, which were then converted into electron volts (eV) (1J = 6.25 × 10^18^ eV).

(6) $$E=hv\;=\frac{hc}{\lambda }$$

where *E* is the energy of a photon (J), *h* is the Planck constant (6.626 × 10^−34^ J s^−1^), *ν* is the frequency of light (s^−1^), *c* is the speed (3 × 10^8^ m s^−1^), and *λ* is the wavelength (nm).

## 3. Results and Discussion

### 3.1. Efficiency of Air Pollutant Removal Under Various Levels of Relative Humidity and Various Initial Concentrations of Pollutants

Table 3 summarizes the test results, showing that in cases of high relative humidity (RH), the concentration of HCHO was reduced from 1.0 to 0.54 ppm over a period of 12 h, which represents a removal rate of 15.97 ± 0.03%. In cases of low RH, the concentration of HCHO was reduced from 1.0 to 0.44 ppm in the same period of time, representing a removal rate of 32.60 ± 0.09%. In cases of high RH, TVOC concentration was reduced from 3.0 to 2.51 ppm (removal rate of 7.12 ± 0.17%), and in cases of low RH, this was reduced from 3.0 to 2.36 ppm (removal rate of 13.56 ± 0.08%). Water molecules are able to block the partial energy of UVGI with a consequent effect on the removal of organic substances. As a result, UVGI was shown to be more efficient in the removal of HCHO and TVOC in cases of low RH than in cases of high RH.

The initial concentration of pollutants has a direct impact on removal efficiency. UVGI was shown to achieve removal rates of 15.97 ± 0.03% and 18.14 ± 0.36% in cases with high and low concentrations of HCHO, respectively. However, the removal rate of TVOC at high concentration was 7.12 ± 0.17% and 5.93 ± 0.25% for low concentrations. Thus, UVGI was shown to be more efficient in the removal of HCHO at low concentrations and TVOC at high concentrations. Factors other than the distance to the UVGI source affect the effectiveness of UVGI in the removal of air pollutants, including the photon energy of the UVGI as well as the nature of the chemical bonds in the VOCs. Repeated experiments presented an error rate between 0.03% and 0.36% in the HCHO and TVOC removal rates, indicating the good reproducibility of the experiment results.

### 3.2. Efficiency of Air Pollutant Removal Through Long-Term Exposure to UVGI

**Figure 4:**
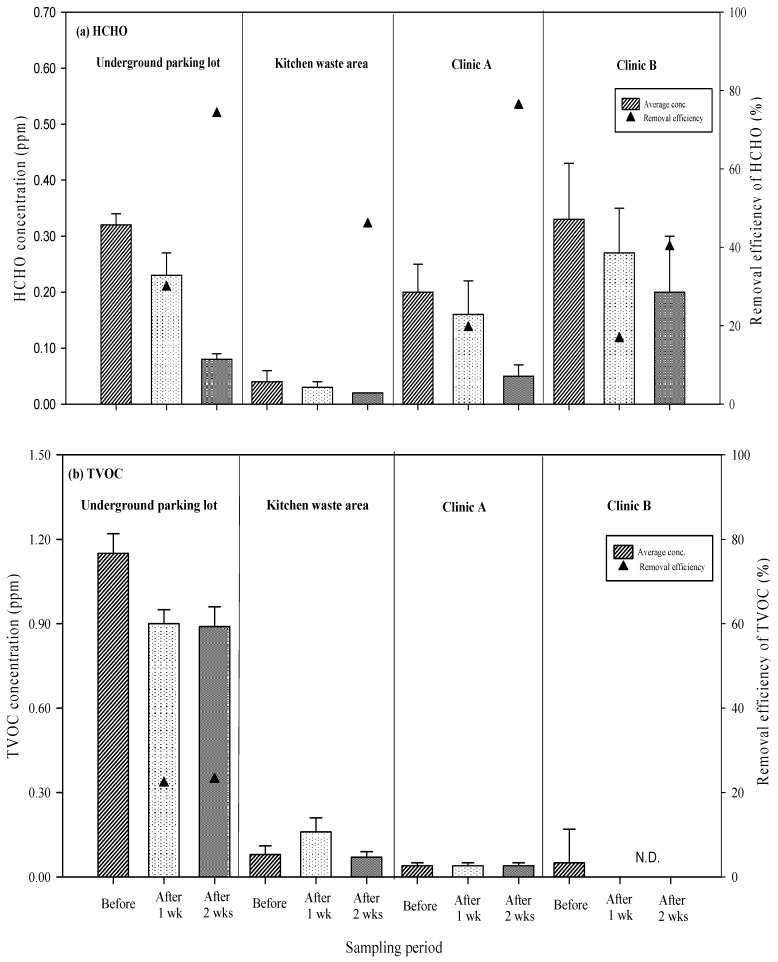
Efficiency of chemical air pollutants: (**a**) HCHO and (**b**) TVOC removal by long-term UVGI exposure.

The efficiency of chemical air pollutant removal by long-term exposure to UVGI is presented in Figure 4a,b. The background concentration of HCHO was lowest in the area used for kitchen waste, presenting an average concentration of 0.04 ppm (0.01–0.06 ppm). After one week of UVGI, the average concentration of HCHO measured 0.03 ppm (0.03–0.04 ppm), indicating a removal rate of 17.1%. After two weeks of irradiation, the average concentration of HCHO declined to 0.02 ppm, representing a removal rate of 45.9%. The indoor background concentration of HCHO was relatively low; therefore, the subsequent removal rates were insignificant. The average background concentrations of HCHO were 0.20 to 0.33 ppm in the underground parking lot, Clinic A, and Clinic B. The high concentrations of HCHO in the underground parking lot were due to the incomplete combustion of organic substances in the exhaust emissions of motor vehicles. Poor ventilation at the site exacerbated the accumulation of pollutants [28,29].

In the clinics, the fumes produced by materials used in building renovation, as well as the volatile medical sterilizers used in the clinics, produced considerable quantities of VOCs. After one week of UVGI, the average concentrations of HCHO in the underground parking lot, Clinic A, and Clinic B measured 0.16 to 0.27 ppm, which represent removal rates of 16.7 to 29.8%. After two weeks, the average concentrations of HCHO declined to 0.05–0.20 ppm, increasing the removal rates to 40.1–76.2%.

With the exception of the underground parking lot, all of the background concentrations of TVOC were low. After one week of UVGI treatment in the parking lot, the average concentration of TVOC measured 0.90 ppm (0.84–0.95 ppm), representing a removal rate of 22.2%. After two weeks of irradiation, the average concentration of TVOC decreased to 0.89 ppm (0.81–0.96 ppm), for a removal rate of 23.1%. According to the experiment result shown, after 12 hours, the average natural decline rate is 9.8% and refers to the results of Angus Shiue et al. [30], the TVOC removal efficiencies of air cleaner. The natural decay ratio is 10.1% and the actual removal efficiency is 83.9%–85.5%. Therefore, this study assumes that the effects of natural decline can be omitted. In Clinic A and Clinic B, the average concentration of TVOC after UVGI irradiation for two weeks (<0.001–0.04 ppm) was close to the background concentration (0.04–0.05 ppm), indicating that the amount of pollutants removed was insignificant. After one week of irradiation, the average concentration of TVOC was 0.16 ppm (0.13–0.21 ppm), which was higher than the background concentration (0.08 ppm, 0.05–0.11 ppm). After the second week, the results were still close to the background concentration (0.07 ppm, 0.06–0.09 ppm). We postulate that these poor removal rates were due to the fact that this study did not focus on a single VOC.

The composition of TVOC varies from site to site. The photon energy (4.89 eV) produced by the UVGI irradiation in this study is sufficient to break single-bond molecules such as in C-C or C-H, but not O-O, as the energy of their bonds range from 65.0 to 119.1 kcal/mol (equivalent to 2.8 to 5.2 eV). The photon energy of UVGI is also insufficient to break the chemical bonds of multiple-bond molecules such as C=O, C=C, and C≡C (6.3 to 8.7 eV). According to Kuo et al. [31] and Kim et al. [32], the primary constituents of VOCs from motor vehicle exhaust are toluene, benzene, xylene, and ethylbenzene. The molecular structures of these chemical substances comprise mainly C-H bonds, which require a minimum wavelength of 289.7 nm (equivalent to 4.3 eV) to promote breakage. The photon energy of the UVGI in this study (4.89 eV) was higher than that required to break C-H bonds; therefore, the removal efficiency of TVOC was higher in the underground parking lot.

The efficiency of long-term exposure to UVGI in the removal of HCHO and TVOC was determined by comparing HCHO readings with background concentrations. After one week of UVGI irradiation, removal rates ranged from 17.1–29.8%, whereas two weeks of UVGI irradiation produced removal rates ranging from 40.1–76.2%. After the second week of UVGI, the removal rates of HCHO measured 23.4–56.7% higher than those of the first week. Formaldehyde removal by UVGI irradiation is associated with the amount of UVGI energy received by the bonds (HCHO + hv → H + HCO•). The molecular formula of HCHO indicates that the C-H and C=O bonds require 98.7 kcal/mol and 176.0 kcal/mol of energy to break. This corresponds to maximum wavelengths of 289.7 nm and 162.4 nm [33], which can be respectively converted into 6.862 × 10^−19^ J and 1.223 × 10^−18^ J of photon energy using the Planck equation, and are equivalent to 4.3 eV and 7.6 eV of energy. The wavelength of the UVGI in this study was 253.7 nm, which is equivalent to 4.89 eV, which is higher than the energy present in the C-H bonds in HCHO (98.7 kcal/mol; 4.3 eV). Thus, direct photolysis is able to break the C-H bonds but not the C=O bonds. As a result, the UVGI in this study was able to remove some, but not all, of the HCHO.

After one week of UVGI irradiation, the concentration levels of TVOC in the kitchen waste area, Clinic B, and Clinic A were either greater than or equal to the background concentrations. Only the underground parking lot displayed a positive removal rate of 22.2%. After two weeks of UVGI, the underground parking lot, kitchen waste area, Clinic A, and Clinic B displayed TVOC removal rates of 11.0 to 100%, demonstrating the effectiveness of UVGI at all four sites. 

**Figure 5:**
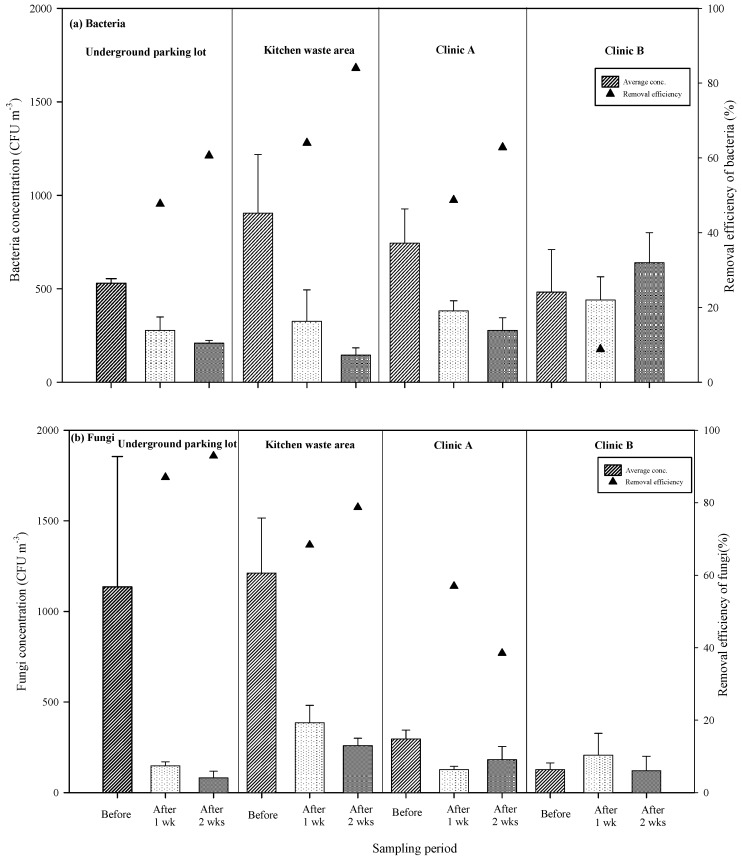
Efficiency of microbiological air pollutants: (**a**) Bacteria and (**b**) fungi removal by long-term UVGI exposure.

The respective average background concentrations of microbiological air pollutants in the underground parking lot, kitchen waste area, Clinic A, and Clinic B are presented in Figure 5a,b. After one week of UVGI, the average concentrations of bacteria measured between 277 CFU m^−3^ and 440 CFU m^−3^, which indicate removal rates of 8.8 to 64.0%. After two weeks, the average bacteria concentrations measured between 145 and 639 CFU m^−3^, which indicates removal rates of −32.7 to 84.0%. With the exception of Clinic B, the removal rates of bacteria all ranged between 47.7% and 84.0%.

After one week of UVGI irradiation, the average concentrations of fungi dropped significantly to 127–385 CFU m^−3^, which indicates removal rates of 57.0 to 87.0% in the underground parking lot, the kitchen waste area, and Clinic A; however, no significant effects were observed in Clinic B. Two weeks of UVGI irradiation lowered the average concentrations of fungi at the four sites to 81–259 CFU m^−3^, representing removal rates of 4.8 to 92.9%. These results indicate that, with the exception of Clinic B, the fungi removal rates at all the sites ranged between 38.5% and 92.9%. The lack of effective sterilizing at Clinic B may be due to the particular strains of microorganisms at that site and/or the FCU ventilation system. The efficiency with which UV light can remove microorganisms depends on the UV dosage, irradiation time, the type of microorganism, its sensitivity to UV light, and how long the microorganisms remain within the UV irradiated area. In order to find out the unusual condition of the Clinical B, the experiment extended the irradiation time and performed the test. The results showed that the bacteria concentration fluctuated at Clinic B; at the 2nd and 4th week, the bacteria concentration was increased (639 CFU/m^3^, 618 CFU/m^3^), but at the 1st, 3rd, 6th week, the bacteria concentration was decreased (440 CFU/m^3^, 370 CFU/m^3^, 215 CFU/m^3^). Based on the above results, it can be inferred that the test time of Clinic B is spring (February to March), for in spring many people of Taiwan are susceptible to dermatitis or skin irritation caused by pollen, dust, and other substances.

With regard to the long-term efficiency of UVGI for removing bacteria and fungi, one week of UVGI reduced the background concentrations of bacteria by 8.8 to 64%, whereas two weeks of UVGI decreased the background concentrations by 60.6 to 84.0% in the kitchen waste area, underground parking lot, and Clinic A. With the exception of Clinic B, the removal rates after the second week of UVGI were 12.9 to 20.0% higher than those of the first week. The poor sterilization at Clinic B may be because the strains and forms of microorganisms present at that site are less sensitive to UVGI [6]. One week of UVGI decreased the background concentrations of fungi by 57.0 to 68.3% and two weeks resulted in removal rates ranging between 4.8% and 92.9%. The rate of fungi removal in the underground parking lot and the kitchen waste area increased by 5.9 to 10.4% after the second week. However, in the clinics, we failed to observe any increase with time. Furthermore, the underground parking lot and the kitchen waste area were more humid that the clinics. Hydration and re-hydration can alter protein structures, thereby influencing the enzymes and nucleic acids involved in DNA repair. The hydration of biopolymer cell walls also moderates the influence of relative humidity on the sterilization effects of UVGI [34].

The effects of UVGI on the removal of bacteria and fungi differed slightly from those reported by Memarzadeh et al. in 2010 [35]. The cell walls of fungal spores are rigid structures, markedly different from the cell walls of prokaryotic bacteria. The DNA in the proteins of thick inner layers of chitin or cellulose can render fungi more resistant to UV light, such that higher UV doses are required for sterilization [36]. In clinic B, UVGI was shown to be inefficient in the removal of bacteria and fungi, perhaps due to the use of mechanical ventilation (a fan coil unit). The type of indoor ventilation and location of intake and exhaust ports can have a significant influence on the vertical mixing of air [23]. UVGI irradiation in Clinic A exhibited good removal efficiency with regard to bacteria but very poor removal efficiency when dealing with fungi. Open windows and doors can influence the movement of aerosols and the primary source of the fungi was the outside environment; therefore, the increase in indoor concentrations can be attributed to swift airflow preventing microorganisms from being sufficiently exposed to UVGI [37,38].

### 3.3. Efficiency of Air Pollutant Removal Using Various UVGI Irradiation Methods

**Figure 6:**
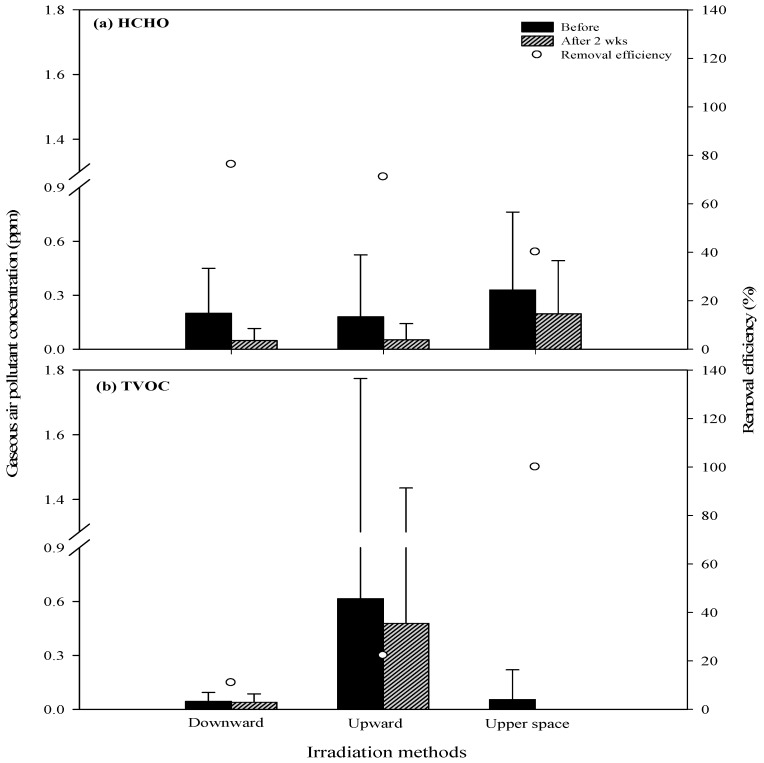
Efficiency of chemical air pollutants: (**a**) HCHO and (**b**) TVOC removal by varying UVGI irradiation methods.

HCHO and TVOC were removed using the UVGI irradiation methods shown in Figure 6a,b. Direct irradiation overnight was the most effective approach to HCHO removal, followed by upward irradiation. Upper space irradiation proved the least effective. Direct irradiation overnight for two weeks reduced background concentrations of HCHO by 76.2% (from 0.20 to 0.05 ppm), while upward irradiation reduced HCHO by 71.7% (from 0.18 to 0.05 ppm). Upward irradiation for two weeks reduced the background concentration of HCHO by 40.1% (from 0.33 to 0.20 ppm). Starting with a TVOC background concentration of 0.05 ppm (<0.001–0.17 ppm), upper space UVGI irradiation for two weeks resulted in the total elimination of TVOC, representing a removal rate of 100%. Upward irradiation for two weeks reduced TVOC background concentrations by 22.26% (from 0.62 to 0.48 ppm). Direct irradiation overnight for two weeks resulted in TVOC background concentrations falling negligibly from between 0.04 and 0.05 ppm to 0.04 ppm (0.03–0.05 ppm). UV photons can break C-C bonds and degrade organic substances; however, the composition of VOCs in indoor air tend to be complex. Good removal efficiency can only be achieved if the indoor TVOC have bonds that UV photons are capable of breaking. Furthermore, the efficiency with which air pollutants are removed by UV light also depends on the UV irradiation time, the UV intensity, and the mixing of air [39].

**Figure 7:**
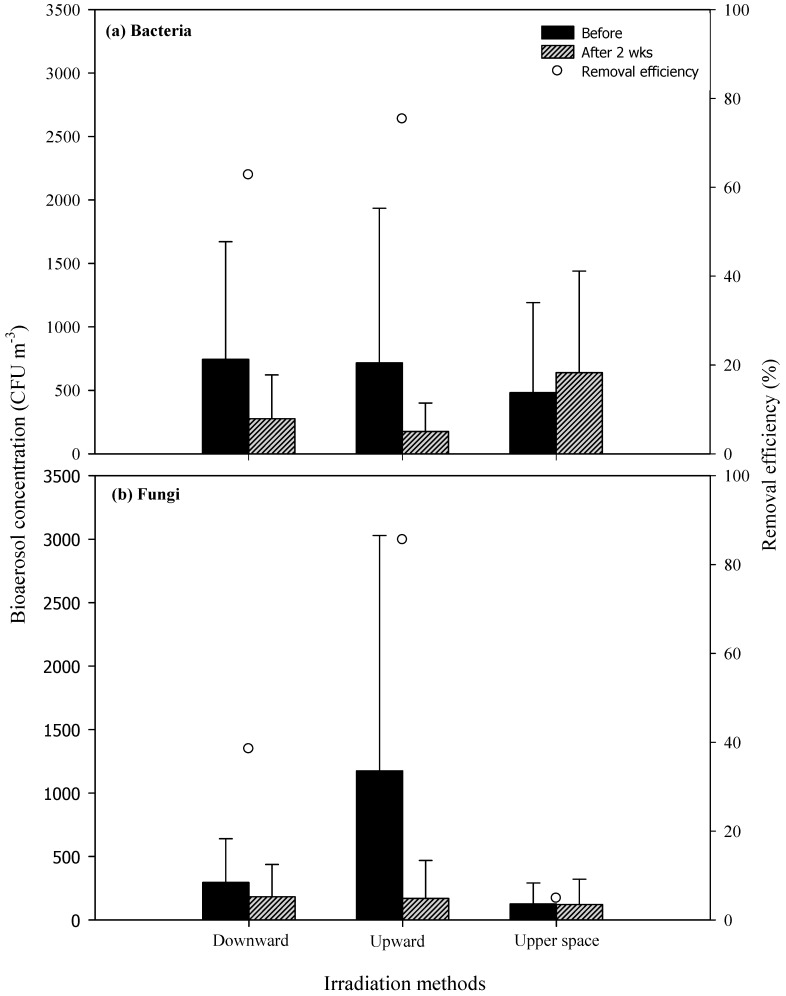
Efficiency of microbiological air pollutants: (**a**) Bacteria and (**b**) fungi removal by varying UVGI irradiation methods.

Upward irradiation proved the most effective at removing microbiological air pollutants (Figure 7a,b), followed by direct irradiation overnight and upper space irradiation. Upward irradiation for two weeks reduced the background concentrations of bacteria from 716 CFU m^−3^ (476–1218 CFU m^−3^) to 177 CFU m^−3^ (111–224 CFU m^−3^) and that of fungi from 1174 CFU m^−3^ (444–1855 CFU m^−3^) to 169 CFU m^−3^ (61–300 CFU m^−3^), representing removal rates of 75.3% and 85.6%, respectively. Direct irradiation overnight for two weeks reduced the background concentration of bacteria from 744 CFU m^−3^ (564–927 CFU m^−3^) to 277 CFU m^−3^ (218–345 CFU m^−3^) and that of fungi from 296 CFU m^−3^ (255–345 CFU m^−3^) to 182 CFU m^−3^ (127–255 CFU m^−3^), representing removal rates of 62.8% and 38.5%, respectively. Direct irradiation overnight for two weeks reduced the background concentration of bacteria from 482 CFU m^−3^ (236–709 CFU m^−3^) to 639 CFU m^−3^ (436–800 CFU m^−3^) and that of fungi from 127 CFU m^−3^ (55–164 CFU m^−3^) to 121 CFU m^−3^ (36–200 CFU m^−3^), which were higher than or equal to the background concentrations. Sterilization involves a number of factors, including the ventilation rate, the intensity of UV irradiation, the physiology and species of the bacteria, the airflow distribution, the relative humidity, and photoreactivity [8]. Our results are consistent with the findings of Brickner et al. from 2003 [24], which showed that bacteria are easier to eliminate than fungi. However, the UVGI dosage required for sterilization varies considerably according to the microorganisms and single-stranded nucleic acids tend to be more sensitive to the effects of UV light than are double-stranded nucleic acids [6].

With regard to the removal of HCHO or TVOC, there are no observable differences in the three UVGI methods (Table 4). In the removal of bacteria, upward irradiation (*p*-value: 0.031) and direct irradiation overnight (*p*-value: 0.027) proved significantly more efficient than upper space irradiation. In the removal of fungi, upward irradiation is more efficient than direct irradiation overnight (*p*-value: 0.007). These findings are consistent with those of Miller and MacHer [4]. A closer distance to the ceiling enables narrowband UVGI to kill the biological PMs carried to the upper space by upward airflow [40]. Direct irradiation overnight and upward irradiation are more direct than upper space irradiation, with regard to the removal of air pollutants. The inefficiency of upper space irradiation may be due to the fact that the UVGI source is placed within the FCU system in the ceiling, which makes air mixing, particularly vertical air mixing, a critical factor. Poor convection in the indoor airflow can prevent air pollutants from being transported to the UV irradiation area to be eliminated [41,42].

## 4. Conclusions

This study tested the efficiency of UVGI in the removal of HCHO and TVOC at various concentrations and under conditions with different levels of relative humidity. Our results indicate that removal efficiency is higher when dealing with low concentrations of HCHO than when dealing with higher concentrations. When dealing with TVOC, removal efficiency is higher when concentrations are higher. Removal efficiency of both HCHO and TVOC is better in conditions of low humidity. Relative humidity produced greater fluctuations in the removal rates than did the initial concentrations of pollutants. Under conditions of high relative humidity, water molecules can provide a barrier to UVGI irradiation, thereby weakening its ability to break down organic compounds.

The application of UVGI for one week resulted in HCHO removal rates ranging from 17.1 to 29.8%, while treatment for two weeks resulted in removal rates between 40.1% and 76.2%. This represents an increase of between 23.4% and 56.7%. No effects were apparent in the UVGI treatment of TVOC after the first week; however, the effect produced noticeable results after the second week. One week of UVGI treatment produced bacteria removal rates between 8.8% and 64%, whereas two weeks resulted in removal rates ranging from 60.6 to 84.0% (with the exception of Clinic B). The removal rates after the second week of UVGI were 12.9 to 20.0% higher than those of the first week. After one week of UVGI, the fungi removal rates ranged between 57.0% and 68.3%, and after two weeks, the removal rates ranged between 4.8% and 92.9%. Therefore, the removal rates of fungi in the underground parking lot and the kitchen waste area only increased by 5.9 to 10.4% after the second week, but those in the medical establishments did not increase. As assumed from the experimental results, the on-site ventilation conditions were controlled to keep the same natural and forced ventilation before and after applying UVGI. During the long-term test period, therefore, the decrease of HCHO and TVOC is due to the UVGI, not by the ventilation.

No significant differences were observed in the removal rates of HCHO or TVOC, such that the background concentrations of air pollutants were lower or close to the concentrations obtained after UV irradiation. Upward and direct irradiation overnight methods were shown to be considerably more efficient in the removal of bacteria than upper space irradiation. Upward irradiation was more efficient in the removal of fungi than direct irradiation overnight. A closer distance to the ceiling made it possible for the narrowband UVGI to kill the biological PMs carried into the upper space by upward airflow.

## Figures and Tables

**Table 1:** Details of building information and UVGI application for field studies.

Case	Parking Lot	Kitchen Waste Area	Clinic A	Clinic B
Building age (year)	3	3	>15	<6 months
Volume (m^3^)	322	756	250	80
Number of population	<2	0	10–15	10–15
Air ventilation type	Mechanical ventilation (exhaust fan)		Natural ventilation	Mechanical ventilation (fan coil unit)
UVGI luminaire	Upward irradiation		Direct irradiation over night	Upper space irradiation
Number of UVGI lamp fixture	8	8	6	6
UVGI lamp intensity	13,800 μW/cm^2^			

**Table 2:** Details of instruments for indoor air quality sampler.

Item	Instrument/Model	Principle	Detection Range	Resolution
HCHO	PPM Technology/PPM Formaldmeter htv-m	Electrochemical	0–10 ppm	0.01 ppm
TVOC	RAE/ppbRAE 3000-10.6 eV	Photo-ionization detector	1ppb–10,000 ppm	1 ppb
Bacteria/ fungi	Thermo/Anderson one-stage sampler	Impacting on agar with incubation (Q: 28.3 LPM)	Stage 0 (8–24 μm) Stage 1 (1–8 μm)	-

**Table 3:** Removal efficiency of duplicate analysis for chemical air pollutants.

Item	Test Condition	High Conc./ Low RH			High Conc./ High RH		Low Conc./ High RH	
		1.0 ppm/40% RH 24 ± 1 °C			1.0 ppm/70% RH 24 ± 1 °C		0.5 ppm/70% RH 24 ± 1 °C	
HCHO	Removal efficiency (%)	#1	#2		#1	#2	#1	#2
		32.54	32.66		15.95	15.99	18.39	17.88
	Avg. ± S.D. (%)	32.6 ± 0.09			15.97 ± 0.03		18.14 ± 0.36	
	Removal of percentage error (%)	−0.36		−0.25			2.77	
TVOC	*Test condition*	*3.0 ppm/40%RH*			*3.0 ppm/70%RH*		*1.4 ppm/70%RH*	
	Removal efficiency (%)	13.61	13.5		7.24	7.00	6.10	5.75
	Avg. ± S.D. (%)	13.56 ± 0.08			7.12 ± 0.17		5.93 ± 0.25	
	Removal of percentage error (%)	4.81		3.31			5.74	

S.D., standard deviation.

**Table 4:** Student’s t-test (two sample unequal variance) for various irradiation methods.

	Air Pollutant	HCHO	TVOC	Bacteria	Fungi
UVGI Luminaires					
Upper space irradiation		0.606	0.212	0.500	0.007 **
Direct irradiation over night		0.103	0.404	0.031 *	0.035
Upward irradiation		0.109	0.268	0.027 *	0.244

* *p*-value < 0.05. ** *p*-value < 0.01.
